# Comment on “Associations of semaglutide with first‐time diagnosis of Alzheimer's disease in patients with type 2 diabetes: Target trial emulation using nationwide real‐world data in the US”

**DOI:** 10.1002/alz.70035

**Published:** 2025-05-12

**Authors:** Amin Salehi‐Abargouei, Taulant Muka, Tinh‐Hai Collet, Kasper P. Kepp, Angeline Chatelan

**Affiliations:** ^1^ Research Center for Food Hygiene and Safety Shahid Sadoughi University of Medical Sciences Alem Square Yazd Iran; ^2^ Department of Nutrition School of Public Health Shahid Sadoughi University of Medical Sciences Alem Square Yazd Iran; ^3^ Yazd Cardiovascular Research Center Institute of Non‐communicable Diseases Shahid Sadoughi University of Medical Sciences Jomhouri Eslami Blvd Yazd Iran; ^4^ Epistudia Schanzenstrasse Bern Switzerland; ^5^ Service of Endocrinology, Diabetology, Nutrition and Therapeutic Education Geneva University Hospitals Geneva Switzerland; ^6^ Diabetes Centre, Faculty of Medicine University of Geneva Geneva Switzerland; ^7^ Department of Nutrition and Dietetics Geneva School of Health Sciences HES‐SO University of Applied Sciences and Arts Western Switzerland Carouge Switzerland

**Keywords:** Alzheimer's disease, glucose‐lowering medications, methodological bias, prevention, semaglutide (Ozempic), type 2 diabetes mellitus

1

We are writing this letter in response to the article by Wang et al. recently published in Alzheimer's & Dementia.^1^ We commend the authors for addressing an important research question using real‐world data; however, substantial methodological limitations affecting the observed benefits of semaglutide (Ozempic®) over other glucose‐lowering medications need to be mentioned.

First, medications for type 2 diabetes mellitus (T2DM) should always be personalized based on medical history and personal preferences targeting glycemic control, as well as cardioprotection, nephroprotection, and other comorbidities.^2^ For instance, semaglutide usually is (i) not considered a first‐line therapy of T2DM and (ii) not prescribed to patients with underweight and/or experiencing weight loss (because it reduces body weight) or those with gastrointestinal conditions. Furthermore, the relationship between body mass and dementia risk is complicated as it probably depends on the nature and severity of the divergent body mass. Underweight in mid‐ and late‐life (and therefore weight loss in middle or older age) might be associated with a higher risk of dementia, whereas late‐life overweight could reduce this risk in some contexts.^3^ Additionally, insulin is usually prescribed when (i) the other glucose‐lowering medications are ineffective, and/or (ii) hyperglycemia is severe, especially if catabolic states like weight loss, hypertriglyceridemia, and ketosis are present.^2^ Therefore, those with uncontrolled diabetes^4^ and with diabetes comorbidities like hypertriglyceridemia,^5^ which are risk factors for dementia, are candidates for insulin therapy and not for semaglutide. In addition, Figure S4 by Wang et al. shows that patients in the semaglutide group had slightly different healthcare encounters compared to the other groups.^1^ In other words, the insulin group is the worst‐off among studied cohorts in a way not fully corrected for. The indication bias related to treatment selection should have been stated in the study limitations and additional analyses controlling for underweight or weight loss should have been added, if possible.

Second, although the authors tried controlling for possible confounders using propensity‐score matching, this method is prone to bias if confounders are not all taken into account.^6^ This seems likely because electronic health records (EHRs) rarely provide valid measures of diet and physical activity, which may be important confounders.^7^ Table 1 suggests this as less than 7% of patients treated with semaglutide or insulin were identified as having “problems with lifestyle,”^1^ while lifestyle measures should be actively reinforced before semaglutide prescription. This percentage is likely underestimated knowing that poor diet and lack of physical activity are key determinants of T2DM and its complications. The same caveat applies to the recording of socioeconomic determinants in EHRs; in many countries patients who use semaglutide have better socioeconomic status due to its high cost. These limitations should have been stated in the paper. In addition, it would be interesting to see and compare the incidence of Alzheimer's disease (AD) between the semaglutide and other glucose‐lowering medications groups in crude and multivariable‐adjusted models, without using propensity‐score matching.

Third, the progression of dementia or AD is very slow, age‐triggered, and may be delayed by many years, relative to onset, with substantial heterogeneity.^8^ People with early‐onset AD have increased healthcare utilization from 5 to 10 years prior to diagnosis^9^ and at least 5 years of follow‐up are recommended for AD prevention trials.^10^ However, Wang et al. observed a difference in AD incidence between the semaglutide and the other glucose‐lowering medications groups already in the first month in some comparisons and little effect after 700 days (≈2 years) (Kaplan–Meier plots, Figure 2B).^1^ Such “early effects” usually suggest a selection bias or missing confounders and, therefore, support our concern that the patient groups are not fully comparable. We believe that a sensitivity analysis by removing those diagnosed with AD in the first year (even 2 years) of the follow‐up would help to estimate the longer‐term effects of semaglutide.

Overall, the article by Wang and colleagues^1^ has several methodological limitations that might affect the suggested benefits of 40 to 70% reduced risk of AD if T2DM is treated with semaglutide. While the drug is surely of interest for the treatment of dementia, we need more studies that adjust for high risks of: (i) selection bias due to treatment strategies selection; (ii) residual confounding by important socioeconomic, body mass, physical activity, and dietary confounders; and (iii) reverse causation due to short follow‐up period, and possible delay in AD diagnosis. Implementing the additional suggested analyses and avoiding using causal language due to the study design could bring new insights into the findings and strengthen confidence in them.

## AUTHOR CONTRIBUTIONS

Amin Salehi‐Abargouei wrote the first draft of the manuscript with Angeline Chatelan. Taulant Muka, Tinh‐Hai Collet, and Kasper P. Kepp reviewed and critically revised the draft. All authors read and approved the final manuscript.

## CONFLICT OF INTEREST STATEMENT

The authors have no conflicting interests to declare. Author disclosures are available in the Supporting Information.

## Supporting information



Supporting Information
